# Impaired dopamine release in Parkinson’s disease

**DOI:** 10.1093/brain/awad064

**Published:** 2023-03-02

**Authors:** Kaitlyn M L Cramb, Dayne Beccano-Kelly, Stephanie J Cragg, Richard Wade-Martins

**Affiliations:** Oxford Parkinson’s Disease Centre and Department of Physiology, Anatomy and Genetics, University of Oxford, Oxford, OX1 3QU, UK; Aligning Science Across Parkinson's (ASAP) Collaborative Research Network, Chevy Chase, MD, 20815, USA; Kavli Institute for Nanoscience Discovery, University of Oxford, Oxford, OX1 3QU, UK; Oxford Parkinson’s Disease Centre and Department of Physiology, Anatomy and Genetics, University of Oxford, Oxford, OX1 3QU, UK; Oxford Parkinson’s Disease Centre and Department of Physiology, Anatomy and Genetics, University of Oxford, Oxford, OX1 3QU, UK; Aligning Science Across Parkinson's (ASAP) Collaborative Research Network, Chevy Chase, MD, 20815, USA; Oxford Parkinson’s Disease Centre and Department of Physiology, Anatomy and Genetics, University of Oxford, Oxford, OX1 3QU, UK; Aligning Science Across Parkinson's (ASAP) Collaborative Research Network, Chevy Chase, MD, 20815, USA; Kavli Institute for Nanoscience Discovery, University of Oxford, Oxford, OX1 3QU, UK

**Keywords:** dopamine release, Parkinson’s disease, neurodegeneration

## Abstract

Parkinson’s disease is the second most common neurodegenerative disease and yet the early pathophysiological events of the condition and sequences of dysfunction remain unclear. The loss of dopaminergic neurons and reduced levels of striatal dopamine are descriptions used interchangeably as underlying the motor deficits in Parkinson’s disease. However, decades of research suggest that dopamine release deficits in Parkinson’s disease do not occur only after cell death, but that there is dysfunction or dysregulation of axonal dopamine release before cell loss. Here we review the evidence for dopamine release deficits prior to neurodegeneration in Parkinson’s disease, drawn from a large and emerging range of Parkinson’s disease models, and the mechanisms by which these release deficits occur. The evidence indicates that impaired dopamine release can result from disruption to a diverse range of Parkinson’s disease-associated genetic and molecular disturbances, and can be considered as a potential pathophysiological hallmark of Parkinson’s disease.

## Teaser

Cramb *et al.* review the evidence for dopamine release deficits prior to neurodegeneration in Parkinson’s disease. They highlight the mechanisms by which these deficits occur and propose areas requiring further investigation.

## Introduction

**Figure 1:**
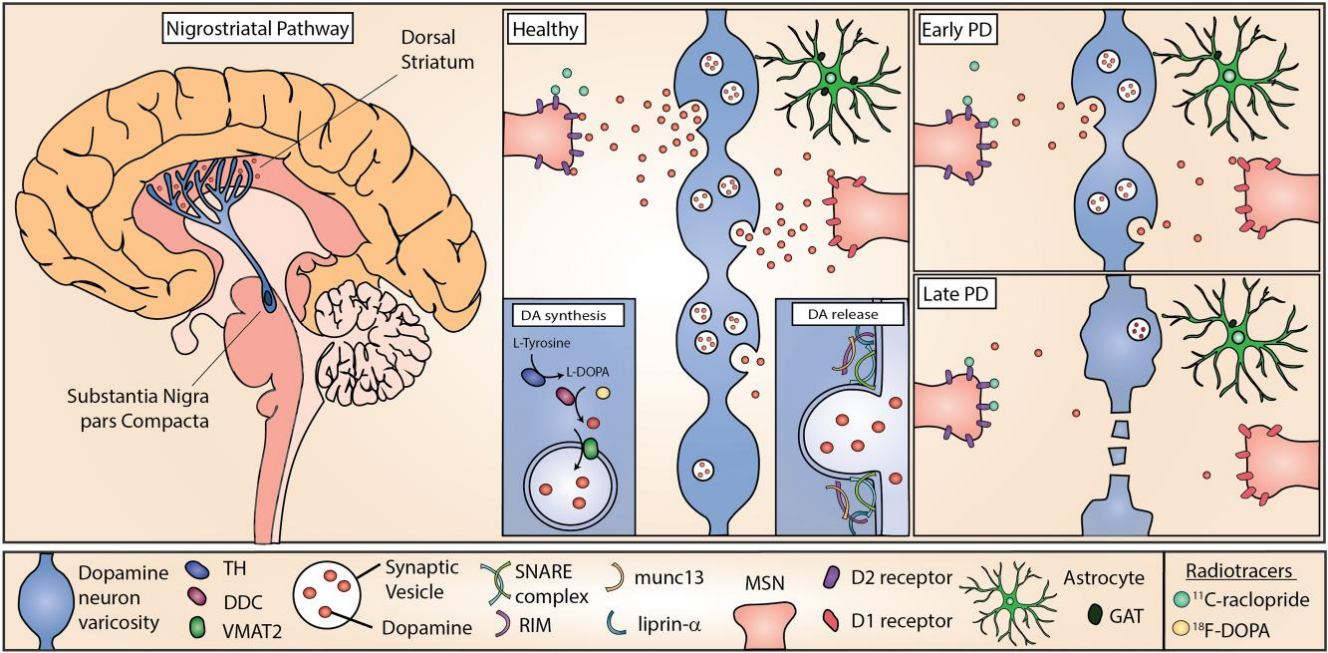
**DA release in the nigrostriatal pathway and simplified working hypothesis for Parkinson’s disease progression.** DAN cell bodies reside in the SNpc and project extensive axonal arbourizations to the dorsal striatum where they release DA (*left*). DA release occurs at active axonal varicosities and acts on D1 and D2 receptors on striatal cells (*middle*). In Parkinson’s disease, the nigrostriatal pathway is most affected and results in progressively decreased DA release (*top right*), and eventually axon degeneration and cell death (*bottom right*) impairing downstream signalling and movement modulation. Cellular dysfunction and altered striatal DA release precede cell death. DA release is modulated by neighbouring cell types including astrocytes, which can contribute to DA release defects observed in Parkinson’s disease models, such as by downregulation of GAT. Radiotracers used to image dopaminergic dysfunction in living human brains include ^11^C-raclopride that binds available D2 receptors and ^18^F-DOPA, which is a substrate for DOPA decarboxylase (DDC).

Parkinson’s disease is one of the most common movement disorders, with debilitating motor symptoms associated with age-related progressive neurodegeneration. Although ∼10% of Parkinson’s disease cases can be linked to inherited monogenic mutations, collectively referred to as familial Parkinson’s disease, most cases, which are referred to as idiopathic or sporadic, have undetermined origin.^1^ The subpopulation of dopamine (DA)-producing neurons of the substantia nigra pars compacta (SNpc) in the midbrain are preferentially affected (Fig. 1).^2,3^ SNpc neurons project to the dorsal striatum forming the nigrostriatal pathway and contribute to basal ganglia circuits involved in action selection, modulation and learning. As hallmarks of Parkinson’s disease, the death of dopaminergic neurons (DANs) and reduction of striatal DA are frequently referred to interchangeably in reports of Parkinson’s disease studies. However, much work in the last decade indicates that DA deficits may not result solely from cell death. A large body of work has demonstrated that in many models of Parkinson’s disease, deficits in DA release from nigrostriatal neurons are present without, or before, neurodegeneration (Fig. 1).

Animal models and, more recently, induced pluripotent stem cell (iPSC)-derived human neuronal models of monogenic Parkinson’s disease, have been useful for understanding disease pathogenesis and functional changes in DANs that might contribute to Parkinson’s disease.^4^ Beyond changes to DA release, these changes include defects in autophagy, intracellular trafficking, mitochondria function and calcium homeostasis.^5–7^ However, there is no unifying hypothesis or single convergent mechanism explaining the onset of Parkinson’s disease to connect early cellular deficits to late-stage Parkinson’s disease motor symptoms. In this review, we discuss DA release defects in Parkinson’s disease models as an early and major pathological feature of the disease.

### The neurobiology of dopamine release

DA release is mediated by Ca^2+^ and Rab3-interacting molecule-dependent vesicular exocytosis from active zone-like sites.^8^ In contrast to the conventional synapse, released DA is thought to act beyond individual synapses at extrasynaptic receptors on many cells and can also be released from the cell body and dendrites through somatodendritic release.^9^ DA release is highly regulated through a variety of mechanisms, including DA synthesis, cell and vesicular uptake, action potential propagation and local neuromodulatory receptors.^10^ DA is synthesized in the cytoplasm in consecutive reactions by the rate-limiting enzyme tyrosine hydroxylase (TH) and DOPA decarboxylase (DDC), coupled with its loading into synaptic vesicles (SVs) by vesicular monoamine transporter 2 (VMAT2) (Fig. 1). The mechanisms through which DA vesicles are handled remain incompletely defined but might differ from many seen at other synapse types. For example, the synaptic molecular machinery for exocytosis from axons involves at least priming and release site scaffolding by Rab3-interacting molecule, munc13 and liprin-α, but might differ from other synapses in requirements for many other active zone proteins.^11^ The biogenesis/recycling pathway of DA SVs involves adaptor protein3 (AP-3) rather than AP-2 that regenerates many other SVs.^12^

DA release dynamics *in vivo* are thought to be determined by a combination of different DAN firing rates (tonic low frequency pacemaking, sustained increases in firing rate and short intermittent or ‘phasic’ bursts at high frequencies), as well as by a range of mechanisms operating on DA axons that can locally gate action potential propagation, DA release probability and its dynamics during activity.^10,13^ DA acts through D1- or D2-type receptors on recipient cells that probably include all striatal cells. Following its release, DA is primarily taken back up by DANs via the DA uptake transporter (DAT). It can also be degraded by monoamine oxidase B or catechol-*O*-methyltransferase.

SNpc DANs have distinctive axonal arbours formed by extensive branching of long unmyelinated axons^14^ that bear exceptionally large numbers of release sites (>10^5^).^15^ Their propagation of action potentials has been proposed to pose extremely high energetic demands.^16^ These factors are suggested to contribute to their preferential vulnerability to degeneration in Parkinson’s disease.^16^ Early synaptic dysfunction in Parkinson’s disease has long been reported as a key feature in both Parkinson’s disease models and human patients.^17^ Dopaminergic axons are affected early in Parkinson’s disease, with synaptic decay preceding neuron death.^18,19^ This concept is not unique to Parkinson’s disease; impaired synaptic activity and function, referred to as synaptopathy, is observed in a wide variety of neurodegenerative diseases, including Huntington’s, Alzheimer’s and motor neuron disease.^18,20^ Although many neurotransmitter pathways are affected in Parkinson’s disease, and DA deficits are frequently considered a relatively late but characteristic pathology of the disease, a substantial body of work suggests DA dysfunction is an early cardinal feature.

### Evidence for early synaptic dysfunction and dopamine release defects in patients with Parkinson’s disease

Much evidence indicates that nigrostriatal degeneration begins in axons,^17^ but is it preceded by DA release defects? Subjects who are at risk for Parkinson’s disease, such as non-manifesting carriers of Parkinson’s disease-associated mutations, asymptomatic family members or individuals with prodromal symptoms, represent the best opportunity to study preclinical Parkinson’s disease and therefore to elucidate systems of early dysfunction.^21^ Brain imaging studies in humans *in vivo* suggest presynaptic defects precede symptom onset in Parkinson’s disease.^17,19,22,23^ PET imaging of asymptomatic family members revealed presynaptic dopaminergic dysfunction, with reduced ^18^F-DOPA uptake, a measure of presynaptic dopaminergic integrity or DA handling, in individuals who later went on to develop Parkinson’s disease.^24^^18^F-DOPA uptake was also significantly reduced in non-manifesting twins that were discordant for Parkinson’s disease.^25,26^ Of these twin pairs, several asymptomatic monozygotic cotwins with abnormal baseline scans later went on to develop clinical Parkinson’s disease.^26^ Similar findings were observed in familial Parkinson’s disease-associated asymptomatic mutation carriers.^23,27,28^ Two studies demonstrated that ^18^F-DOPA uptake was reduced in asymptomatic carriers with a single parkin (*PRKN*) mutation allele when compared to a control cohort.^23,28^ A 5-year follow-up study suggested that although subclinical reductions of striatal ^18^F-DOPA uptake are frequent in single parkin mutation carriers, the rate of disease progression appeared very slow, although confirmation with a larger and longer-term longitudinal follow-up study, would be required.^29^ Asymptomatic individuals heterozygous for PTEN-induced kinase 1 (*PINK1*) mutations also demonstrated a significant reduction in ^18^F-DOPA uptake when compared to a control cohort.^30^ Studies demonstrate that in asymptomatic Y1699C and R1441C leucine-rich repeat kinase 2 (*LRRK2)* mutation carriers, ^18^F-DOPA uptake was normal, despite evidence for impaired DA function with abnormal DAT binding.^27,31^ A longitudinal study that followed asymptomatic *LRRK2* G2019S carriers who went on to convert to Parkinson’s disease by the time of a 4-year re-evaluation revealed a lower striatal DAT binding at baseline than nonconverters.^32^ Brain imaging of patients with prodromal Parkinson’s disease, including hyposmic and REM sleep behaviour disorder cohorts, has also revealed dopaminergic dysfunction.^21,22^ Combined, these studies suggest there is probably presynaptic dopaminergic dysfunction in Parkinson’s disease-affected individuals that precedes Parkinson’s disease onset.

Although imaging methods are useful to measure DA dynamics in living human brains, most techniques use indirect measurements of DA levels and none has yet directly measured DA release in early parkinsonism. For example, PET measurements of ^11^C-raclopride, which competes with DA to bind to D2 receptors, may indeed report on differences in DA release. However, these measurements fundamentally report on D2 receptor availability, and so may also represent alterations in D2 receptor expression, integrity of postsynaptic projections or loss of DA from degeneration of presynaptic terminals. Likewise, differences in ^18^F-DOPA measurements, a marker of presynaptic DA storage capacity, could indicate a change in the number of dopaminergic cells, but may also be influenced by alterations in DA metabolism and handling in intact neurons. Further, most of the studies performed on prodromal, preclinical or genetic carriers involve small sample sizes, are not longitudinal and rather rely upon comparisons to a nominal control cohort. Incomplete penetrance of many Parkinson’s disease-associated mutations means that truly preclinical subjects cannot be confirmed without long-term follow-up. Therefore, although DA release may be defective before degeneration in Parkinson’s disease patients, limitations in technology have made the study of DA release in humans challenging. Animal and human cell-based models of Parkinson’s disease have been useful in closing this gap.

## Dopamine release in animal and human-based cell models of Parkinson’s disease

Many animal and human cell-based models of Parkinson’s disease have demonstrated early synaptic dysfunction and intrinsic DA release defects. These have been reported in models that have recapitulated Parkinson’s disease-related SNpc cell death and motor deficits, as well as in models that do not. The tools available to study these models of Parkinson’s disease, such as fast-scan cyclic voltammetry (FCV), allow for opportunities to investigate DA dynamics directly, in ways not readily possible in the inaccessible human brain.

In this section, we address the evidence for DA release defects in animal and human-based cell models of major monogenic forms of autosomal dominant (*SNCA*, *LRRK2* and *VPS35*) and autosomal recessive [*PRKN* (parkin), *PINK1* and *PARK7* (DJ-1)] familial Parkinson’s disease and those carrying the major genetic risk factor for Parkinson’s disease onset (*GBA1*). We focus here on genetic forms of Parkinson’s disease as although toxin-based models offer a powerful approach to understand the impact of DAN loss on circuit function, the rapid loss of neurons caused by toxins does not allow for an analysis of early and progressive neuron dysfunction preceding cell death in Parkinson’s disease.

### Autosomal dominant genes

#### *SNCA* (PARK1)

Both familial and sporadic Parkinson’s disease have been linked to aberrant levels of the α-synuclein protein, encoded by the gene *SNCA*. Heterozygous missense mutations or multiplications of *SNCA* cause severe early-onset Parkinson’s disease. α-synuclein has multiple critical roles at the presynaptic site including binding to negatively charged phospholipids and interacting with several synaptic proteins.^33–37^

Several α-synuclein Parkinson’s disease mouse models that recapitulate nigrostriatal neuronal loss and motor deficits have reported DA release defects consistently before neurodegeneration (Table 1). In a bacterial artificial chromosome transgenic (BAC Tg) mouse model that expressed human α-synuclein at disease-relevant levels, and which displayed age-dependent parkinsonian phenotypes, DA neurotransmission defects were observed.^51^ Reduction in evoked DA release *ex vivo*, restricted to the dorsal striatum, was detected by FCV from a young age, without loss of striatal DA content and prior to the loss of DANsand the onset of motor symptoms.^51^ Viral overexpression of human α-synuclein in the rat substantia nigra resulted in reduced evoked striatal DA release detected using amperometry *in vivo* and *ex vivo* before the loss of DANs, axonal fibres and reduced DA content^52,53^ but that corresponded to motor deficits.^52^ In addition, an increase in extracellular DA concentration was detected using microdialysis in a transgenic mouse model overexpressing human wild-type α-synuclein under the Thy1 promoter, before a later change in striatal DA content and motor deficits, and without cell death.^54^ Further, a mouse model expressing truncated human α-synuclein with an enhanced ability to aggregate exhibited KCl-evoked DA release defects using microdialysis.^70^ These defects are not limited to animal models; DA release defects were observed in iPSC-derived DANs from patients carrying the *SNCA* triplication.^71^

**Table 1:** Dopamine release defects in Parkinson’s disease models

Transgene	Model	DA release defect?	Cell death?	Major motor symptoms?	Reference
**Autosomal dominant**					
LRRK2	Human R1441G BAC transgenic mouse	Decreased release	None	Present	Li *et al.*^38^
	R1441G BAC transgenic mouse	None	Not investigated	Not investigated	Sanchez *et al.*^39^
	R1441C and G2019S BAC transgenic rat	Decreased release	None	Present	Sloan *et al.*^40^
	G2019S BAC transgenic mouse	Decreased release	None	None	Li *et al.*^41^
	Human BAC G2019S mouse	Decreased release	None	None	Melrose *et al.*^42^
	G2019S KI mouse	Decreased release	None	None	Yue *et al.*^43^
	Midbrain-specific (PitX3) overexpression of G2019S mouse	Decreased release	None	None	Liu *et al.*^44^
	G2019S KI mouse	None	None	Not investigated	Longo *et al.*^45^
	G2019S KI mouse	Increased release	Not investigated	Not investigated	Volta *et al.*^46^
	iPSC-derived DANs with I2020T	Decreased release	n/a	n/a	Ohta *et al.*^47^
	iPSC-derived DANs with I723V and M23497T	Decreased release	n/a	n/a	Luo *et al.*^48^
VPS35	D620N KI mouse	Decreased release	None	None	Ishizu *et al.*^49^
	D620N KI mouse	Increased release	None	None	Cataldi *et al.*^50^
SNCA	BAC human SNCA overexpression mouse	Decreased release	Present	Present	Janezic *et al.*^51^
	A30P and SNCA overexpression AAV rat	Decreased release^a^	None	Present	Gaugler *et al.*^52^
	AAV human SNCA rat	Decreased release	Present	Not investigated	Lundblad *et al*.^53^
	SNCA overexpression mouse	Increased release	Present	Present	Lam *et al.*^54^
	Midbrain-specific (PitX3) expression of A53T mouse	Decreased release	Present	Present	Lin *et al.*^55^
	Human A53T overexpression mouse	Altered release	Not investigated	Not investigated	Platt *et al.*^56^
	A30P BAC transgenic mouse	Decreased release	None	None	Taylor *et al.*^57^
	Human A30P mouse	Decreased release	None	Present	Yavich *et al.*^58^
**Autosomal recessive**					
Parkin	Parkin KO mouse	Decreased release	None	None	Oyama *et al.*^59^
	Parkin KO mouse	Decreased release	Not investigated	Not investigated	Kitada *et al.*^60^
	Parkin KO mouse	Decreased release	None	Present	Goldberg *et al.*^61^
	Parkin KO mouse	None at 6-8 weeks	Not investigated	Not investigated	Sanchez *et al.*^39^
	Parkin KO rat	None	None	None	Creed *et al.*^62^
	Parkin KO iPSC-derived DANs	Increased release	n/a	n/a	Jiang *et al.*^63^
Pink1	Pink1 KO mouse	Decreased release	None	Not investigated	Kitada *et al.*^64^
	Pink1 KO mouse	Decreased release	None	Not investigated	Zhi *et al.*^65^
	Pink1 KO mouse	None	Not investigated	Not investigated	Sanchez *et al.*^39^
	Pink1 KO mouse	Increased release	Present	Present	Creed *et al.*^62^
DJ-1	DJ-1 KO rat	None	Present	Present	Creed *et al.*^62^
	DJ-1 KO mouse	Decreased release	None	Present	Goldberg *et al.*^66^
	DJ-1 KO mouse	None	Not investigated	Not investigated	Sanchez *et al.*^39^
	DJ-1 KO mouse	None	None	Present	Chandran *et al.*^67^
**Risk factor**					
GBA	Wild-type mice with chronic conduritol-β-epoxide treatment	Decreased release	Not investigated	Present	Ginns *et al.*^68^
	iPSC-derived DANs with N370S	Decreased release	n/a	n/a	Woodard *et al.*^69^

Decreased release in SNCA overexpression but not SNCA A30P.

Changes to DA transmission have also been reported in models expressing Parkinson’s disease-related missense mutations in *SNCA*. In a mouse model inducibly overexpressing the *SNCA-A53T* Parkinson’s disease mutation in PITX3-expressing midbrain DA neurons, severe decreases in basal and evoked DA release *in vivo* and evoked DA release *ex vivo* were observed, preceding widespread neuron degeneration and major motor defects.^55^ Overexpression of human A53T α-synuclein under the mouse prion promoter resulted in more subtle evoked release defects *ex vivo* using FCV, with modified frequency-dependent release and enhanced recovery following prolonged stimulation.^56^ In a BAC Tg mouse model expressing the Parkinson’s disease-associated *SNCA-A30P* mutation, a reduction of evoked DA release, but not norepinephrine, in the dorsal but not ventral striatum *ex vivo* was observed at an early age (3–4 months) in the absence of overt cell death or loss of DA content^57^ while another mouse model expressing human *SNCA-A30P* under the control of a prion promoter resulted in a decrease of evoked DA release *in vivo* in the absence of loss of DA at 12–14 months^58^ but with locomotor defects.

Although *SNCA* genetic mutation or overexpression models inducing α-synuclein aggregation may provide useful information on how aggregation can affect neuronal dysfunction, *SNCA* genetic alterations that induce aggregation may be very rare, or are not naturally occurring Parkinson’s disease-associated mutations in patients, such as α-synuclein truncation. Genetic models may not, therefore, represent disease aetiology relevant to sporadic Parkinson’s disease that is associated with α-synuclein aggregation in the absence of *SNCA* mutations. Recent advances have applied injection of α-synuclein preformed fibrils (PFFs) into the brain to generate protein aggregates as a model of Parkinson’s disease. Aggregation of α-synuclein modelled in this way can induce DA release defects in the absence of genetic mutations or increased α-synuclein expression levels. Specifically, DA release defects have been observed following the injection of α-synuclein PFFs into the SNpc or striatum of wild-type rats or mice, or transgenic mice expressing human α-synuclein.^72–74^

Injection of the wild-type α-synuclein PFFs to generate an aggregate pathology that may be considered ‘Lewy body-like’ has been proposed as a route to model sporadic Parkinson’s disease in animals. Models employing SNpc or intrastriatal PFF injections that take advantage of the connectivity to the SNpc have the advantages that they have been shown to aggregate endogenous α-synuclein, induce progressive DA system neurodegeneration specific to SNpc while sparing the ventral tegmental area and recapitulate Parkinson’s disease hallmarks, such as motor system deficits.^75^ However, methods of preparation of PFFs, such as the protein concentration used and aggregate subtype morphology obtained, as well as delivery strategies and injection sites, can lead to inconsistent results.^76,77^ Nevertheless, these localized approaches have rapidly gained in popularity as a model to generate a progressive pathology useful to understand Parkinson’s disease in the context of dysfunction caused by α-synuclein aggregation, and efforts have been extended to standardize practices to reduce inconsistencies.^78^ The α-synuclein PFF and oligomer treatment models have also been extended to iPSC-derived DANs allowing findings to be repeated in human DANs derived from Parkinson’s patients.^79–81^ Why most Parkinson’s patients have α-synuclein aggregation in the absence of mutations in *SNCA* remains a central unknown question in Parkinson’s disease. Understanding the relationship between the formation of α-synuclein aggregates and deficits in DA release in dysfunctioning, but living, neurons is critical to understanding the disease aetiology of sporadic Parkinson’s disease.

Do DA release deficits reflect gains or losses in the normal function of α-synuclein? When α-synuclein is knocked out, several studies report dysfunctional neurotransmitter release that is generally the opposite of α-synuclein overexpression or mutation.^82–85^ While a decrease in the recoverability of subsequently evoked striatal DA release was reported in one knockout mouse model of α-synuclein,^84^ other studies have reported no change to evoked striatal DA release levels.^82^ However, double knockout of both α and γ synuclein, and triple knockout of all three synucleins, α, β and γ (SynTKO) results in increased evoked striatal DA release *ex vivo*.^82,86^ A recent study of α-synuclein knockout and SynTKO mice revealed a more complex role of α-synuclein on DA release using *in vivo* FCV, with roles in facilitation and depression of DA release dependent on specific patterns of neuronal activity.^85^ Combined, these studies support a gain-of-function of α-synuclein leads to DA dysfunction in Parkinson’s disease, however, both increased and decreased alterations in α-synuclein levels can have severe effects on several aspects of DAN biology and it remains to be ascertained which is causative for DA release defects in Parkinson’s disease models.

#### *LRRK2* (PARK8)

Autosomal dominant gain-of-function mutations in *LRRK2* result in the most common form of familial Parkinson’s disease.^87^*LRRK2* encodes a large protein of 2527 amino acids and is known to have roles in autophagy, endocytosis and various protein interactions.^87^ Evidence suggests Parkinson’s disease-related *LRRK2* mutations lead to DA release defects.

In several rodent models overexpressing either the G2019S or R1441C/G mutations of LRRK2, decreased age-dependent evoked and basal DA release were reported using microdialysis or FCV (Table 1).^38–40^ This decrease in release was observed in the absence of impaired motor function or overt midbrain neurodegeneration, with the exception of Liu *et al*.^44^ who reported axonal degeneration only in older aged mice with midbrain-specific LRRK2-G2019S expression, and both Li *et al*.^38^ and Sloan *et al.*^40^ who observed motor defects in their BAC Tg models at older ages. None of these models exhibited SNpc DAN death. Notably, one study reported an earlier increase in evoked DA release using FCV *ex vivo* in 3-month-old animals expressing *LRRK2-G2019S* that resolved by 12 months.^46^ This increase in evoked release was not observed by 8 weeks in *LRRK2-R1441G* mice using FCV *ex vivo*.^39^ Defective release has also been observed in human neurons with Parkinson’s disease mutations. Large decreases in evoked DA release were reported in *LRRK2-I2020T* iPSC-derived DANs measured by high performance liquid chromatography with electrochemical detection (HPLC-ECD),^47^ and *LRRK2-I723V*/*M2397T* iPSC-derived DANs measured by ELISA.^48^ In contrast, despite observing alterations in both VMAT2 and DAT expression, no DA release defect was observed in Lrrk2-G2019S knock-in mice using microdialysis.^45^

Interestingly, DA release does not seem to be affected by *LRRK2* knockout. Studies of *Lrrk2* knockout rats or mice report no decrease in basal or evoked DA release using microdialysis.^62,89^ Striatal DA levels quantified by HPLC are also normal in *Lrrk2* knockout mice.^90,91^ Therefore, although LRRK2 plays a critical role in synaptic activity, a gain but not loss of function is detrimental to DA release.

#### *VPS35* (PARK17)

Vacuolar protein sorting protein 35 (VPS35) is a component of a retromer complex involved in membrane-associated protein trafficking including synaptic receptor D1.^92,93^ Although rare, disease-causing missense mutations result in autosomal dominant Parkinson’s disease clinically indistinguishable from idiopathic Parkinson’s disease.^94^

Reports have demonstrated that DA release is dysfunctional in models carrying Parkinson’s disease-associated mutations in *Vps35*. Knock-in mice carrying the Parkinson’s disease-associated *Vps35-D620N* mutation have a significant reduction in evoked DA release by microdialysis in the absence of neurodegeneration and in the presence of normal DA synthesis at 20–28 weeks old (Table 1).^49^ A further *Vps35-D630N* knock-in mouse model was reported to have an early (at 3 months) increase in evoked DA release by FCV.^50^

### Autosomal recessive genes with typical parkinsonism

#### Parkin (PARK2)

Loss-of-function mutations in parkin are the most common cause of autosomal recessive Parkinson’s.^95^ Parkin, encoded by the *PRKN* gene (previously known as *PARK2*), is an E3 ubiquitin ligase that regulates proteasome-dependent protein degradation and plays a critical role in mitochondrial quality control. Although mainly connected to Parkinson’s disease through its role in mitochondrial dysfunction, evidence suggests that loss-of-function parkin mutations lead to DA release defects.

Homozygous parkin knockout mice have decreased evoked DA release between 2 and 6 months of age using *in vivo* voltammetry^59^ and *ex vivo* single-pulse amperometry,^60^ but interestingly, no difference at 9 and 12 months.^59^ In contrast, increased basal DA release was reported using *in vivo* microdialysis at later ages (8–9 months) in the absence of changes of DA content in a homozygous parkin knockout mouse.^61^ A further study on a homozygous parkin knockout rat reported no evoked DA release deficit using *in vivo* microdialysis, although did observe an increase in glycine release and significant decreases in DA metabolite levels reflecting alterations in DA metabolism.^62^ No deficit was observed before 8 weeks in parkin knockout mice using *ex vivo* FCV.^39^ IPSC-derived mature DANs from Parkinson’s disease patients with exon deletions in parkin display a significant increase in evoked DA release using HPLC.^63^

#### *PINK1* (PARK6)

PINK1 is a mitochondrial protein kinase in which loss-of-function mutations are the second most frequent known cause of autosomal recessive Parkinson’s disease.^96^ This protein regulates the activity of parkin, affects mitochondrial stability and modulates mitophagy; however, several studies have reported evidence for Parkinson’s disease mutations in PINK1 resulting in DA release defects in both animal and human-based models before or without neuronal cell death.

Knockout of *Pink1* results in an age-dependent decrease in evoked DA release using FCV *ex vivo* with no effect at 3–4 months but a 30% decrease at ∼12 months of age in the absence of neurodegeneration.^65^ Decreased evoked DA release at early ages has been reported in some models using amperometry *ex vivo*,^64^ but not in others using single-pulse FCV.^39,65^ In rats, knockout of *Pink1* results in an age-dependent alterations in evoked release of several neurotransmitters including DA and acetylcholine measured by microdialysis.^62^

#### DJ-1 (PARK7)

DJ-1 is a ubiquitously-expressed multifunctional protein best known as a modulator of reactive oxygen species (ROS), protecting cells from oxidative stress through self-oxidation and initiating an anti-oxidant stress response.^97^ Genetic deletions and point mutations resulting in loss of activity of this protein are implicated in autosomal recessive Parkinson’s disease.^98^

Studies addressing DA release in animal models with genetic deletions of DJ-1 seemingly contradict. One study reported no effect on evoked striatal DA release in DJ-1 knockout rats using microdialysis but, however, did report age-dependent changes in acetylcholine and glutamate release and, importantly, the long time-scale of the experiment may have masked more subtle evoked release defects.^62^ Similarly, in DJ-1 knock out mice, no KCl-evoked DA release defects were observed at 6 months of age measured by microdialysis^67^ or before 8 weeks using FCV.^39^ By contrast, another study reported decreased evoked release using FCV in DJ-1 knockout mice in the absence of neurodegeneration.^66^ Importantly, this study reported pronounced locomotor defects in this model.

### Major risk factors for Parkinson’s disease

#### GBA1

*GBA1*, which encodes the enzyme glucocerebrosidase (GCase), a lysosomal enzyme involved in sphingolipid degradation, is the most common strong genetic risk factor for Parkinson’s disease.^99^ While homozygous mutations in this gene result in the lysosomal storage disorder Gaucher’s disease, heterozygous mutations are connected to increased risk for Parkinson’s disease.^99^ Exactly how *GBA1* mutations increase risk and whether this can be attributed to loss- or gain-of-function mechanisms remains under debate. Nevertheless, models have provided evidence that Parkinson’s disease-associated mutations in *GBA1* are connected to synaptic dysfunction.

Subchronic pharmacological inhibition of GCase causes nigrostriatal dopaminergic dysfunction in mice, producing a substantial evoked DA release deficit using *in vivo* microdialysis.^68^ Expression of the Parkinson’s disease-associated mutation *GBA-L444P* results in increased susceptibility to striatal DA deficits as measured by HPLC following neurotoxin treatment compared to non-mutant GBA.^100^ IPSC-derived neurons have been used to investigate DA release defects in monozygotic twins harbouring a heterozygous *GBA-N370S* mutation and clinically discordant for Parkinson’s disease.^69^ Reduced release of DA was observed using HPLC in cells from the affected twin. Of note, neurons from one control with a family history of Parkinson’s disease had reduced DA release compared to other control neurons without a family history of Parkinson’s disease. Combined, these results indicate that Parkinson’s disease-related mutations may lead to subtle DA release deficits that can be aggravated by non-genetic factors. Investigations into the factors that lead to discordant phenotypes and aggravated DA release defects will be important not only to understand *GBA1*-related Parkinson’s disease, but also for understanding sporadic disease pathogenesis.

## Inconsistent dopamine release phenotypes

Studies detailing opposite trends in dysfunctional DA release are not necessarily contradictory and might be explained by a variety of differences, such as in the techniques used. Most of the studies discussed use either FCV or microdialysis coupled with HPLC-ECD.^8^ FCV is capable of reporting on sub-second and region-specific measurements of dynamic changes in DA release, and is particularly well suited to quantify the concentrations of discretely evoked DA release as well as reuptake dynamics in *ex vivo* brain slices, typically coupled with local stimulation. In contrast, microdialysis is typically used to sample extracellular DA over longer timescales that will represent integrated values of tonic and phasic release. As these techniques differ profoundly in their temporal and spatial resolution, and often in use of local stimulations, it is no surprise they can report inconsistent phenotypes. For example, there may be alterations in DA reuptake, DA neuron firing rates or local circuit integration that obscure differences in DA release when sampled on a longer time-scale. In addition, seemingly opposing phenotypes may be also be explained by differences in ages sampled.

## Mechanisms of dopamine release defects

**Figure 2:**
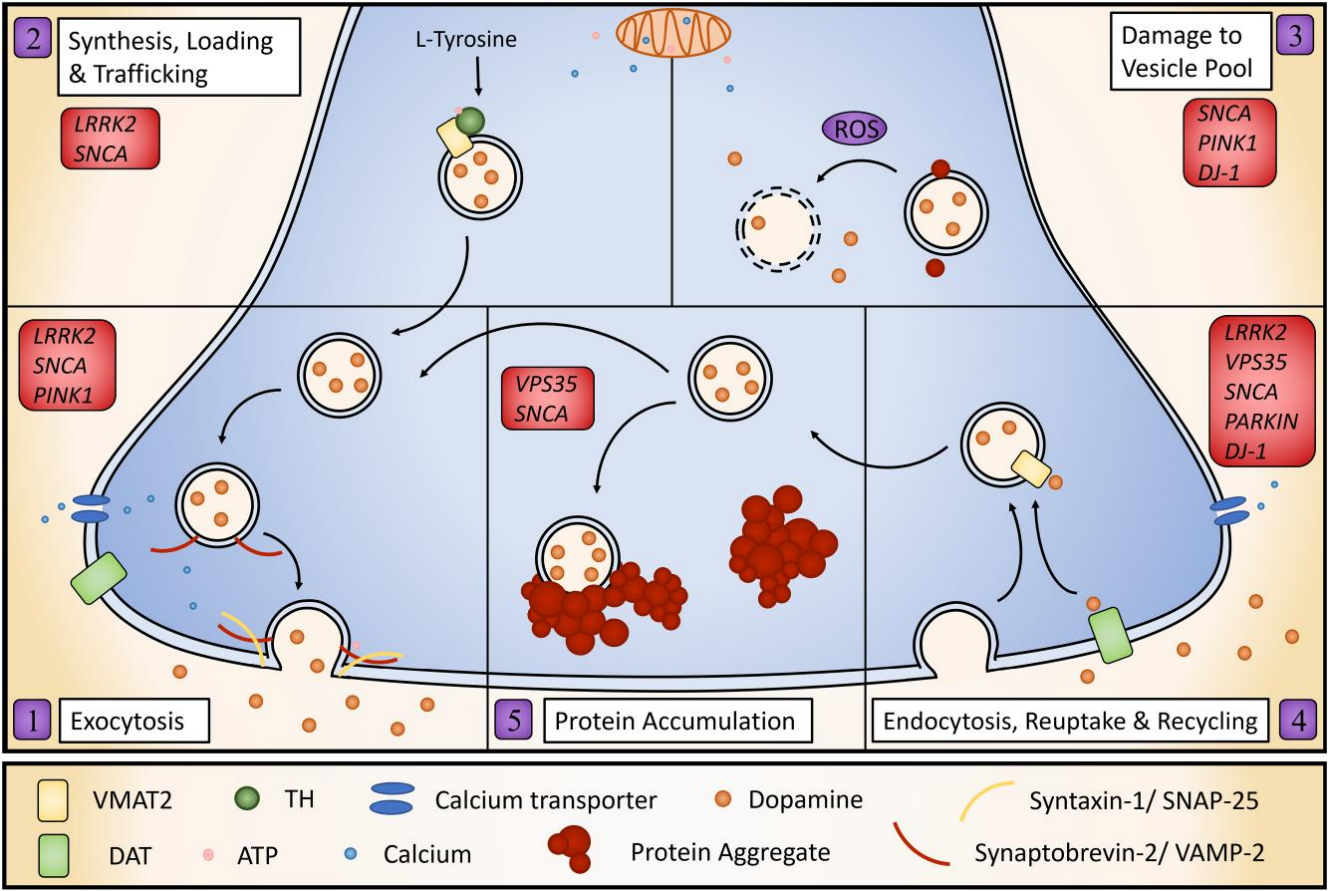
**Cell-autonomous presynaptic mechanisms of dysfunctional DA release in Parkinson’s disease models.** Defects in many different aspects of DA handling can lead to defective DA release. These include: defective exocytosis machinery including SNARE-complex formation and fusion (1); impaired DA synthesis, its loading into SVs via VMAT2 and trafficking to release sites (2); damage to the vesicular pool (3); recycling of DA following release, including recycling of SV through endocytosis and reuptake via DAT (4); and protein aggregation (5).

It is evident that multiple Parkinson’s disease-associated proteins with diverse cellular roles converge on synaptic defects in Parkinson’s disease.^101^ These defects have been repeatedly shown to result in DA release deficits and the mechanisms by which these occur can be categorized into six mechanisms as discussed next: SV exocytosis, SV trafficking and loading, SV endocytosis and recycling, synaptic protein accumulation, damage to the SV pool and non-cell autonomous mechanisms (Fig. 2).

### Synaptic vesicle exocytosis

Most DA release is mediated by Ca^2+^-dependent vesicular exocytosis^8^ and disruption to this process will cause impairment in DA release (Fig. 2). Normal synaptic functioning in DA axons probably requires the formation of the SNARE complex by vesicular synaptobrevin-2/VAMP-2 and syntaxin-1/SNAP-25 on the plasma membrane. Parkinson’s disease proteins such as α-synuclein and LRRK2 directly interact with SNARE proteins and Parkinson’s disease-related mutations either alter activity or expression of SNARE complex.^47,102–104^ For example, LRRK2-dependent phosphorylation of Snapin inhibits SNAP25 and results in the loss of readily releasable pool, impairing exocytosis.^102^ Further, the addition of α-synuclein oligomers inhibits SNARE-complex formation by binding SNARE protein, synaptobrevin-2.^105^ Exocytosis also requires high levels of energy in the form of mitochondrial ATP.^106^ Parkinson’s disease mutations and α-synuclein oligomers can impair mitochondrial function or transport leading to an axonal energy deficit and synaptic loss.^81,107^ Models of many Parkinson’s disease mutations including *Pink1* and *SNCA* have reported impaired ATP production.^7,65,108,109^ Defective mitochondria may also disrupt exocytosis through calcium dysregulation, as these organelles are critical for calcium buffering.^107^ Calcium dyshomeostasis has been reported in many Parkinson’s disease models including those with mutations in *Pink1* and *GBA*.^108,110^ Further, DA release by exocytosis from axons only occurs when the synapse receives appropriate signals; mechanisms that govern axonal excitability are particularly important for governing action potential propagation,^13^ and defects in axonal biophysics will lead to defects in DA release. Many different mechanisms can, therefore, cause defective exocytosis, leading to disrupted DA release in DANs.

### Synaptic vesicle trafficking and loading

DA release can also be affected by unavailability of DA in the readily releasable pool at presynaptic terminals, through defects in synthesis, vesicular loading or trafficking (Fig. 2). Synthesis of DA in the cytoplasm is coupled with its loading into SVs by VMAT2. Parkinson’s disease models with mutations in *LRRK2*, *DJ-1* or *VPS35* have alterations in VMAT2 levels, pointing towards disturbances in SV loading.^45,50,111^ SV loading depends on an electrochemical gradient between the vesicle and cytoplasm, established by vesicular H^+^-ATPase. Alterations in SV pH or H^+^-ATPase activity lead to ineffective VMAT2 activity and quantal size of DA release.^10,112^ Disruption of this pH gradient might also occur through mitochondrial dysfunction as it requires ATP. Loss of DA in the SVs by disturbed synaptic loading not only leads to a lack of availability of DA for release but the consequent increase in cytoplasmic DA may have toxic effects on the cell as it is rapidly converted to a reactive species.^113–115^ Indeed, overexpression of *SNCA* mutation A30P leads to disrupted SV vesicle pH and an increase in cytosolic catecholamines.^116^ Furthermore, intracellular and specifically axonal and vesicular trafficking is impaired in Parkinson’s disease with consequences to synaptic function.^117,118^ Trafficking and organization of SVs from the SV pool to the active site which regulates the size of the readily releasable pool is vital for appropriate DA release.^119^ Synucleins are key regulators of SV pool organization^120^ and models of Parkinson’s disease with *SNCA* mutations have demonstrated this to be impaired, disrupting DA release.^51^ Further, α-synuclein oligomers may disrupt trafficking of SVs as they negatively affect axonal transport.^81^ Reduced SV DA availability may also be caused by disrupted DA synthesis. TH, the rate-limiting enzyme in DA synthesis, has been shown to have decreased protein levels or decreased activity in models of Parkinson’s disease carrying mutations in *LRRK2, SNCA*, *DJ-1* and *PINK1*.^44,111,121,122^

### Damage to the synaptic vesicle pool

Direct damage to the SV pool might also result in a DA release deficit (Fig. 2). This may occur through generation of ROS, a common hallmark of Parkinson’s disease. Beyond supplying ATP and modulating Ca^2+^ levels, mitochondria are also key producers of ROS at the synaptic site.^106^ Models with Parkinson’s disease mutations such as *Pink1*, *DJ-1* and *SNCA* have been connected to increased ROS.^108,123^ Further, cytosolic DA, which is increased in Parkinson’s disease models, undergoes auto-oxidation, creating further ROS. Increased cytosolic DA may be caused by a number of factors, including impaired DA loading into SV or increased DAT activity, both connected to Parkinson’s disease.^45^ Presynaptic ROS signalling has been identified as a key regulator of synaptic neurotransmission.^124^ Overproduction of ROS inhibits SNARE-complex assembly and thus impairs exocytosis.^106^ Indeed, overproduction of mitochondrial ROS induced motor defects by impaired synaptic activity in motor neurons in *Xenopus laevis* tadpoles.^125^ Damage to the SV pool can also occur in other ways. Protofibrillar α-synuclein and oligomers disrupt the SV membrane allowing DA to spill out into the cytosol^126^ and alter neuron excitability.^127^ Further, reactive DA metabolite DOPAL can promote the formation of α-synuclein oligomers and cause damage to SV, particularly by allowing protons to leak out and raise the pH.^128^ Damage to the SV pool may be, therefore, a mechanism by which DA release defects may occur.

### Synaptic vesicle endocytosis, recycling and dopamine reuptake

The ability to reconstitute the vesicular pool for subsequent release events is regulated by vesicle renewal and also reuptake, recycling and reloading of the neurotransmitter into SVs. Functioning as an electrogenic Na^+^/Cl^−^ symporter, the DA transporter, DAT, is the principal mechanism to both terminate neurotransmission and recycle DA to reuse in subsequent cycles. Both genetic deletion and overexpression of DAT decreases DA release through different mechanisms.^113^ Models of Parkinson’s disease with mutations in *SNCA*, *Vps35*, *DJ-1*, parkin and *LRRK2* have reported alterations in DAT levels or function.^46,50,129–131^ Furthermore, tightly regulated endocytosis occurs immediately following exocytosis to retrieve the SV membrane and machinery for further neurotransmission.^132^ Substantial evidence links Parkinson’s disease with defects in SV endocytosis and recycling.^101^ A well-characterized mechanism for the involvement of LRRK2 in defective neurotransmission is by the role it plays in the phosphorylation of RABs, such as Rab5b, key players in SV endocytosis and trafficking.^133^ LRRK2 can also regulate endocytosis and SV recycling through phosphorylation of auxilin, endophilinA and synaptojanin1, and disrupted phosphorylation in models with Parkinson’s disease mutations in LRRK2 leads to synaptic defects.^134^ LRRK2 kinase activity may also influence SV fusion by altering the rate of SNARE-complex disassembly.^135^ This mechanism is not limited to LRRK2; Nguyen *et al.*^101^ discuss the strong evidence for the link between defective endocytosis and several Parkinson’s disease-linked genes including *SH3GL2* (endophilin A1), *DNAJC6* (auxilin*)*, *SYNJ1* (synaptojanin1), *LRRK2*, parkin and *VPS35*. Further to this list, both complete *DJ-1* knockout as well as Parkinson’s disease-related *DJ-1* mutations disrupt SV endocytosis.^136^ Substantial evidence supports that defective SV recycling and decreased reuptake of DA leading to lack of SV replenishment could be an important contributing factor to disruption of DA release in Parkinson’s disease (Fig. 2).

### Protein accumulation in the synapse

Protein aggregation is a major hallmark of Parkinson’s disease. Aggregation of α-synuclein in particular may induce DA release defects by physically blocking cell processes caused by the aggregate itself or rather through sequestration of the functional protein, leading to disruption of synaptic processes by loss of protein function. The addition of oligomeric α-synuclein and PFFs in the absence of genetic alterations results in DA release defects.^72,105^ Further, many models of Parkinson’s disease-related proteins such as LRRK2, PINK1, GCase and VPS35 report increased α-synuclein expression and aggregation^68,137–139^ or decreased degradation^140^ which suggests the pathological mechanisms may occur in many of these models, at least in part, indirectly through α-synuclein. However, disruptive protein accumulation and aggregation is not limited to α-synuclein. Reports have suggested protein turnover machinery may get ‘clogged’ in Parkinson’s disease, thus leading to accumulation of many different proteins and disruption of synaptic homeostasis.^118^ Indeed, altered synaptic protein homeostasis and defective protein turnover is a consistent feature of many Parkinson’s disease models. A major protein degradation pathway, autophagy, is critical to regulating protein turnover in neurons and has been connected to Parkinson’s disease-related mutations including *SNCA*, *LRRK2*, *VPS35*, parkin, *Pink1* and *DJ-1*.^6,141^ Autophagy appears to be specifically important for synaptic homeostasis in DA systems.^118^ Therefore, protein aggregation may disrupt DA release through sequestration of essential proteins or by physical interference in critical processes for release (Fig. 2).

### Non-cell-autonomous mechanisms

Mechanisms of defective DA release may not be limited to axonal dysfunction; modulation of DA release involves other neuronal and non-neuronal networks.^10^ Recent research suggests these neuronal and non-neuronal striatal networks contribute to defective release in Parkinson’s disease.^142^ Specifically, DA release is under tonic inhibition by striatal GABA tone, governed by GABA uptake transporters (GATs) on astrocytes in the dorsolateral striatum. These become downregulated in a mouse Parkinson’s disease model with human SNCA overexpression, leading to enhanced GABAergic inhibition of DA release that will support DA release deficits.^142^ Thus, other networks operating at the level of DA axons might contribute to, or conversely partly mask, defective DA release in Parkinson’s disease.

**Figure 3:**
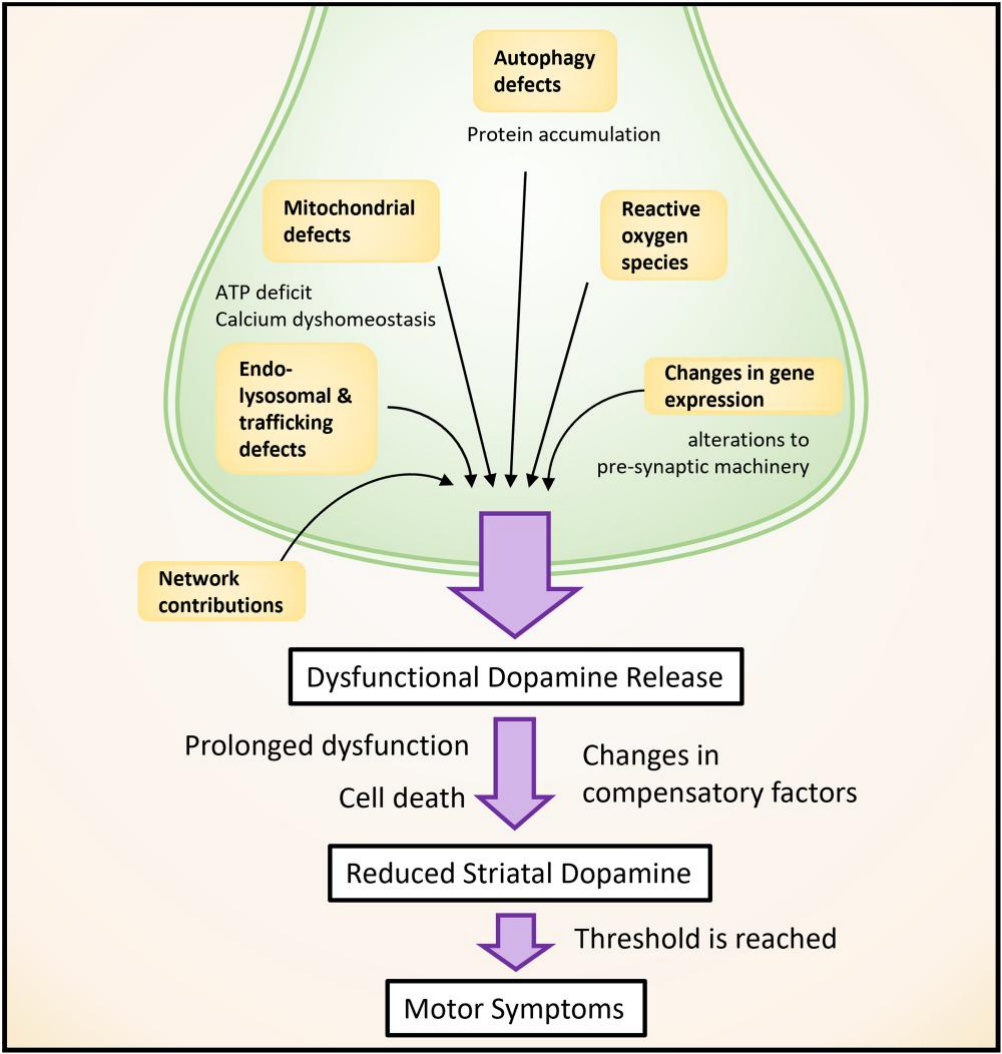
**Overview of multifactorial cellular dysfunction converging on defective DA release**. Dysfunction in several different cellular processes result in dysfunctional DA release in Parkinson’s disease models. Prolonged dysfunction, probably in combination with cell death, leads to reduced striatal DA that, once a threshold is reached, leads to the hallmark motor symptoms of Parkinson’s disease. The level of this threshold is modulated by compensatory factors.

Models of sporadic and familial Parkinson’s disease exhibit dysfunction in the endolysosomal pathway, mitochondrial dysfunction, calcium signalling and autophagy. Although these are well supported by substantial evidence in many models, there is no established explanation for how these mechanisms lead to Parkinson’s disease symptom onset, the loss of striatal DA or neuronal death. We have discussed in this section how each of these affected Parkinson’s disease-associated pathways can disrupt DA release, frequently before the onset of neurodegeneration and motor deficits (Fig. 3). Importantly, many of these affected processes are critical to general cell function, and yet degeneration is only observed in a subset of neurons. The preferential vulnerability of DANs to these forms of dysfunction, resulting in DA release defects, is an explanation that connects broad cellular dysfunction to the major pathological hallmark of reduced striatal DA that results in motor deficits in Parkinson’s disease (Fig. 3).

The fact that DA neurotransmission defects are observed in many different models of Parkinson’s disease in the absence of neurodegeneration supports DA release defects as a precursor to Parkinson’s disease onset. What it does not explain is at what point does symptom onset occur, nor does it explain at what point does cell death begin and, most importantly, whether the former is fundamentally contingent on the latter.

## Is cell death required for the onset of Parkinson’s disease symptoms?

A substantial number of Parkinson’s disease models report ‘failures’ to recapitulate nigrostriatal neuron death. The absence of cell death is widely accepted to be due to species-specific differences, for example, the lifespan of the model. These are nevertheless useful models of prodromal Parkinson’s disease; however, others envisage the ‘failure’ to recapitulate what is considered a fundamental Parkinson’s disease hallmark as an indication of an inadequate model of the disease. As a consequence, animal models that do not demonstrate nigrostriatal cell death have often not been analysed for behavioural changes due to the time-consuming and costly requirements, particularly as, in the working hypothesis, no behavioural alterations would be expected. Despite this, some reports observed motor phenotypes in the absence of cell death in Parkinson’s disease animal models.^38,40,58,61,66,143^ This finding presents a critical challenge to the definition of Parkinson’s disease. Although widely accepted as a key hallmark in Parkinson’s disease, there remains a lack of evidence from human and animal studies for the absolute *necessity* of cell death for the onset of Parkinson’s disease symptoms. If reduced striatal DA release resulting in loss of striatal DA content is commonplace in Parkinson’s disease models, can motor symptoms simply be explained by striatal DA content loss in Parkinson’s disease without requiring neuronal death? A reduction in DA release might even act as a pathophysiological driver of early Parkinson’s disease with motor symptoms occurring if release is sufficiently impaired to reduce striatal DA content past a threshold when it cannot be compensated for by other factors (Fig. 3).

As discussed, DA release defects are routinely observed before motor symptom onset. How much of a deficit is required to produce motor symptoms, and perhaps more importantly, what other factors are modulating striatal DA content levels to either prevent, or contribute to, symptom onset? Reduced DA release does not always directly correspond to reduced striatal DA content at a given time point,^51^ and extracellular levels can be modulated by other factors such as DA reuptake. The absolute DA content threshold required for symptom onset is probably modulated by network and compensatory mechanisms such as upregulation of postsynaptic DA receptor numbers, which has been reported in humans with early Parkinson’s disease.^144^ Importantly, the studies exploring DA release defects in Parkinson’s disease support that it is prolonged dysfunction that is the key to motor symptom onset. On a molecular level, this is probably due to the progression of several aspects of cellular dysfunction coinciding with loss of systemic compensation, however, further studies are still required to fully understand this.

Indeed, neuronal death may be a critical switch, tipping the system beyond the ability to compensate for a system-wide deficit of striatal DA content, leading to the onset of motor symptoms. The mechanism by which neurons die in Parkinson’s disease remains unclear. Synaptic dysfunction, or rather a side effect of it, might contribute to cell death through toxic cytosolic DA or α-synuclein accumulation.^115,145^ On the other hand, this death may be caused by programmed cell death following prolonged cellular dysfunction.^146^ Whether or not neuronal death is the mechanism by which reduced DA release passes the threshold, ultimately it is the loss of DA content itself, rather than the death of neurons, which is required for the onset of motor symptoms.

## Outstanding questions pertaining to defective dopamine release in Parkinson’s disease

The robust evidence for early DA release defects across a broad range of Parkinson’s disease models inspires many avenues of future study. Most of these studies focus on DA and presynaptic mechanisms of release deficits, which is therefore the focus of this review, but evidence suggests there is much more involved. We outline the outstanding questions below that should be a focus of future studies.

First, how do other neuronal and non-neuronal cell types contribute to observed DA release defects? DA release is known to be highly regulated by activity in numerous other networks.^10^ For example, changes to striatal GABA tone contribute to DA release deficits.^142^ Cholinergic interneurons strongly modify DA release and its dynamics,^147^ which can occur locally by acting on the distal dopaminergic axon in the striatum.^148^ Parkinson’s disease is suggested to include diverse modifications to acetylcholine function throughout the brain, but whether striatal DA release defects might perhaps be attributable to dysfunction in cholinergic interneurons, either as a secondary or primary driver, is not well understood. The role of non-DA neurons in DA release is especially of interest for Parkinson’s disease genes such as *LRRK2*, which have relatively low expression in DANs but are enriched elsewhere e.g. in cholinergic interneurons, while still exhibiting DA release deficit phenotypes. The striatum contains a rich neuromodulatory soup whose impact on DA axon function and dysfunction is not well understood, but is an active area of new research. Non-neuronal cell types may also play a critical regulatory role in DA release dysfunction. The DA release deficit in a model of familial Parkinson’s disease attributed to elevated tonic GABA discussed before is thought to arise from GAT downregulation on astrocytes.^142^ Given the role of microglia in synaptic pruning and its connection to neurotransmission defects in neurodegenerative disorders,^149^ there is also potential for microglia to contribute to observed defects in Parkinson’s disease.

Second, how are defects in the release of other neurotransmitters from DANs contributing to disease pathology? Axonal dysfunction in DANs in Parkinson’s disease might not be limited to the release of DA. Co-transmission of neurotransmitters provides a method of regulation and fine tuning of responses within a complex system such as the basal ganglia. This co-transmission by other neurotransmitters released from nigrostriatal DANs is also dysfunctional in Parkinson’s disease models with significant consequences. GABA co-transmission in DANs is decreased early in a model of familial Parkinson’s disease before cell loss,^142^ which might play a role in patient phenotypes. Furthermore, partial DAN loss in a Parkinson’s disease mouse model leads to reductions in glutamate co-transmission from nigrostriatal axons in dorsolateral striatum and a downregulation of glutamate mGluR1 receptors in striatal cholinergic interneurons where, remarkably, targeted re-expression of these receptors can rescue motor deficits.^150^ Combined, these findings identify a potential contribution of defective co-transmission to the pathophysiology of Parkinson’s disease and suggest new opportunities for therapeutic intervention.

Further, several studies report broader synaptic dysfunction in non-dopaminergic systems, implicating a variety of neurotransmitters.^62^ This is consistent with the understanding that Parkinson’s disease is not solely a movement disorder, with pathology not exclusively limited to dysfunction of motor control.^151^ However, it is also well known that the dorsal striatum is particularly vulnerable compared to ventral striatum. Indeed, reports demonstrate specificity of release deficits in rodent models not only to DANs, but also regionally to the dorsal, but not ventral, striatum.^40,51,57^ Understanding the preferential vulnerability of specific networks to dysfunction in Parkinson’s disease while others remain intact has yet to been resolved with many questions remaining around the cell-type specificity of synaptic dysfunction in Parkinson’s disease.

The subcellular location of the defective DA release of a SNpc DAN can produce distinct motor phenotypes. A recent report demonstrated that early parkinsonism is caused by defective DA release by mitochondrial dysfunction preceding cell death.^143^ In a conditional *Ndufs2*-knockout mouse that causes mitochondrial complex I disruption, loss of axonal DA release generated deficits in motor learning and fine motor control but was necessary but not sufficient for full levodopa-responsive parkinsonism, for which impairment of somatodendritic release within the SNpc was required. Interestingly, axonal DA release occurred first, with somatodendric DA release defects following later, which suggests more thorough temporal analyses of DA release defects could reveal subtle but important differences in Parkinson’s disease models. Somatodendritic DA release in the SNpc may play a critical role in Parkinson’s disease pathogenesis and very few studies to date address defective somatodendritic DA release in genetic models of Parkinson’s disease. The recent advances in optical sensors, such as dLight1 and GRAB_DA_^152,153^ allow for higher sensitivity detection of DA, and will allow study of DA release defects throughout a wider range of brain areas than has been permissible previously.

Despite a great deal of evidence for DA release defects in a broad range of animal models of Parkinson’s disease, studies in human cells have been more limited but are ripe for exploration. The development of iPSC-derived DAN technology opens plenty of opportunities to study neuronal dysfunction, and continues to hold great potential for expansion of DA release research. However, exploration of DA release defects in of iPSC-derived DAN monocultures requires the disease pathogenicity to be cell autonomous. Increasing efforts to produce viable neuronal/glial co-cultures and anatomically accurate human midbrain organoids that better recapitulate the connectivity of the neural networks within which DA neurons are connected in the brain, will greatly increase our ability to explore the disease more accurately.^154^

## Closing remarks

Parkinson’s disease is characterized by the degeneration of the nigrostriatal pathway and loss of DANs in the SNpc, typically, amongst a constellation of dysfunctions in multiple systems. Robust evidence collected over several decades supports that DAN pathology manifests first as a dysfunction in DA axons that project to the dorsal striatum. In this review, we have summarized the DA release defects reported in models of Parkinson’s disease and the mechanisms by which they are currently known to occur. These defects frequently precede motor symptom onset and neuronal cell loss and seem to be now well established as a marker of Parkinson’s disease prior to degeneration. Much less well explored is whether in turn, synaptic dysfunction is a marker that only heralds imminent cellular demise as an innocent bystander, or whether, this deficit has a detrimental impact on cell viability that contributes to the disease process, catalysing disease progression through symptom onset or cell death. If DA synapse dysfunction is not simply reporting disease onset, but is supporting disease progression, then future strategies to restore axon function might in turn lead to neuroprotection. An improved understanding of the mechanisms underlying DA release deficits could therefore offer hope not just for improved symptom-treating therapies, but become a focus for the design of early neuroprotective therapeutics.

## Supplementary Material

awad064_Supplementary_Data

## Glossary

**Action potential:** An electrical signal involving the rapid change in membrane potential along a neuronal surface to transport information throughout the body. Upon reaching nerve terminals, action potentials lead to the release of neurotransmitters into the extracellular space to signal to neighbouring neurons.

**Co-transmission**: the release of two or more different neurotransmitters from a single presynaptic site.

**Dopamine:** A catecholamine neurotransmitter present in distinct pathways in the brain and involved in several functions, including motor control and reward-motivated behaviour.

**Dopamine release:** The general term by which dopamine is discharged into the extracellular space by a dopaminergic neuron in a controlled manner, usually following an action potential and by Ca^2+^-dependent exocytosis, for the purpose of signalling to neighbouring neurons.

**Dying back hypothesis:** A prevailing hypothesis in several neurodegenerative diseases that supports that degeneration begins at nerve terminals.

**Familial Parkinson’s disease:** A subset of Parkinson’s disease cases in which the cause of the disease is attributed to a specific inherited genetic mutation.

**FCV:** A type of voltammetry with relatively high spatiotemporal resolution that is commonly used to measure electrochemically active metabolites *in vivo* or *ex vivo*.

**Idiopathic/sporadic Parkinson’s disease:** The majority of Parkinson’s disease cases in which the disease aetiology and cause is unknown.

**Induced pluripotent stem cell (iPSC):** Laboratory-generated stem cells that maintain the genetic information of the donor but have the capacity to differentiate into most cell types. IPSCs are produced by reprogramming somatic tissues such as skin or blood cells by artificially expressing key transcription factors.

**Microdialysis:** A technique used to measure metabolite quantities *in vivo*, by sampling extracellular fluid coupled with quantification using mass spectrometry or HPLC and electrochemical detection.

**Neurodegeneration:** The general term that refers to age-associated progressive loss of function and structure of neurons, often manifesting as cell death. This degeneration leads to debilitating diseases including Parkinson’s, Alzheimer’s and Huntington’s disease.

**Nigrostriatal pathway:** One of the major dopaminergic pathways of the brain. The cell bodies of dopamine-producing neurons reside in the SNpc and project towards the dorsal striatum where they release dopamine.

**Pacemaking activity:** Autonomous activity specific to a small number of neurons in which self-generated action potentials arise independent of other neuronal input signals.

**PET:** A non-invasive neuroimaging technique used to assess physiological function, such as blood flow, oxygen consumption or metabolism and activity of various proteins, with the use of specific radiotracers. PET is often used to diagnosis disease in humans.

**Somatodendritic release:** The process by which some neurons can release neurotransmitters from either the cell body/soma or dendrites in addition to the conventional synaptic release from axons. The mechanism and purpose of somatodendritic release is not fully clear, but is considered a minor component of total neurotransmitter release by the neuron.

**Substantia nigra pars compacta (SNpc):** A structure within the basal ganglia of the midbrain, named for its darker appearance due to the presence of high levels of the pigment neuromelanin. The pars compacta, one of two main components of the SN, forms the main component of the nigrostriatal pathway supplying the dorsal striatum with dopamine.

**Synaptic vesicles (SV):** Phospholipid membrane-bound compartments containing neurotransmitters to be released into the extracellular space for signalling to neighbouring cells, generally across a synaptic cleft.

**Synaptopathy:** A term referring to dysfunction of the synapse.
