# Supplementary material for: Impaired dopamine release in Parkinson’s disease
Source: Brain. 2023 Mar 2;146(8):3117–32. doi: 10.1093/brain/awad064 (PMC10393405; doi:10.1093/brain/awad064)
Supplement: awad064_Supplementary_Data [file awad064_supplementary_data.pdf]

Supplementary Table 1: Dopamine release defects in PD models (extended)

Mutation type	TRANS GENE	MODEL	DA RELEASE DEFECT?	METHOD OF DA DETECTION	CELL DEATH?	MAJOR MOTOR SYMPTOMS?	Ref.
Autosomal dominant	LRRK2	Human R1441G BAC transgenic mouse	**Decreased release**	In vivo **microdialysis** with nomefensine (DAT blocker)	**None** by 9-10 months	**Present** at 6-12 months	*Li, 2009*
		R1441G BAC transgenic mouse	**None** at 6-8 weeks	Single-pulse & pulse-train **FCV**	Not investigated	Not investigated	*Sanchez, 2014*
		R1441C and G2019S BAC transgenic rat	**Decreased release** at 18-21 months	Single-pulse **FCV**	**None** by 18-21 months	**Present** at 18-21 months	*Sloan, 2016*
		G2019S BAC transgenic mouse	**Decreased release** at 12 months	Single-pulse **FCV** in brain slices	**None by** 20 months	None	*Li, 2010*
		Human BAC G2019S mouse	**Decreased** at 8-10 months	In vivo **microdialysis** with amphetamine challenge	**None** by 22-24 months	**None** by 7-8 months	*Melrose, 2010*
		G2019S KI mouse	**Decreased release and basal levels** at 12 months (but not 6 months)	In vivo **microdialysis** with amphetamine challenge	**None** by 20 months	**None** by 12 months	*Yue, 2015*
		Midbrain-specific (PitX3) overexpression of G2019S mouse	**Decreased release** at 12 months but not 1 month	Single-pulse **FCV** in brain slices	**None** by 20 months	**None** by 18 months	*Liu, 2015*
		G2019S KI mouse	**None** at 12 months	In vivo **microdialysis** with 10 and 20 mM KCl	**None** by 19 months	Not investigated	*Longo, 2017*
		G2019S KI mouse	**Increased release** at 3 months	Single pulse **FCV** in brain slices	Not investigated	Not investigated	*Volta, 2017*
		iPSC-derived DANs with I2020T	**Decreased release** at 86 DIV	Evoked with 56 mM KCl coupled to HPLC-ECD	n/a	n/a	*Ohta, 2015*
		iPSC-derived DANs with I723V and M23497T	**Decreased release** at 37 DIV	Evoked with 56 mM KCl detected with ELISA	n/a	n/a	*Luo, 2020*
	VPS35	D620N KI mouse	**Decreased release** by 20-28 weeks	In vivo **microdialysis** with 120 mM KCl	**None** by 70 weeks	**None** by 12 weeks	*Ishizu, 2016*
		D620N KI mouse	**Increased release** at 3 months	Single-pulse **FCV** in brain slices	**None** by 3 months	**None** by 3 months	*Cataldi, 2018*
	SNCA	BAC human SNCA overexpression mouse	**Decreased release** by 3 months and present until atleast 18 months	Single-pulse **FCV** in brain slices	**Present** at 18 months	**Present** at 18 months	*Janezic, 2013*
		A30P and SNCA overexpression AAV rat	**Decreased release** 9 weeks post injection for WT OVX but not A30P	**Amperometry** with 70 mM KCl	**None**	**Present** 4 months post injection (WT overexpression not A30P)	*Gaugler, 2012*
		AAV human SNCA rat	**Decreased release**	In vivo **amperometry** with KCl	**Present**	Not investigated	*Lundblad, 2012*
		SNCA overexpression mouse	**Increased release** early**,** later normalized	In vivo **microdialysis**	**Present** at 14 months of age	**Present** at 14 months of age	*Lam, 2011*
		Midbrain-specific (PitX3) expression of A53T mouse	**Decreased release** at 3-4 months	In vivo **microdialysis** and **FCV** in brain slices	**Present**	**Present,** severe by 12 months	*Lin, 2012*

		Human A53T overexpression mouse	**Altered release** at 6-12 months	**FCV** in brain slices	Not investigated	Not investigated	*Platt, 2012*
		A30P BAC transgenic mouse	**Decreased release**	**FCV** in brain slices	**None** by 18 months of age	**None**	*Taylor, 2014*
		Human A30P mouse	**Decreased release**	**FCV** in brain slices	**None**	**Present** by 6 months	*Yavich, 2005*
**Autosomal recessive**	Parkin	Parkin KO mouse	**Decreased release** between 3 -6months	In vivo **voltammetry**	**None** by 12 months	**None** by 12 months	*Oyama, 2010*
		Parkin KO mouse	**Decreased release** between 8-16 weeks	Single pulse **amperometry**	Not investigated	Not investigated	*Kitada, 2009*
		Parkin KO mouse	**Decreased release**	In vivo **microdialysis**	**None** by 24 months	**Present**	*Goldberg, 2003*
		Parkin KO mouse	**None** at 6-8 weeks	Single pulse **FCV**	Not investigated	Not investigated	*Sanchez, 2014*
		Parkin KO rat	**None**	In vivo **microdialysis** with KCl	**None**	**None**	*Creed, 2019*
		Parkin KO iPSC-derived DANs	**Increased release**	Evoked with KCl coupled to HPLC-ECD	n/a	n/a	*Jiang, 2012*
	Pink1	Pink1 KO mouse	**Decreased release**	**Amperometry**	**None**	Not investigated	*Kitada, 2007*
		Pink1 KO mouse	**Decreased release**	**FCV** in brain slices	**None** by 14 months	Not investigated	*Zhi, 2019*
		Pink1 KO mouse	**None** at 6-8 weeks	Single pulse **FCV** +/- Nomefensine	Not investigated	Not investigated	*Sanchez, 2014*
		Pink1 KO mouse	**Increased release**	In vivo **microdialysis** with KCl	**Present** by 8 months	**Present** by 8 months	*Creed, 2019*
	DJ-1	DJ-1 KO rat	**None**	In vivo **microdialysis** with KCl	**Present** by 8 months	**Present** by 8 months	*Creed, 2019*
		DJ-1 KO mouse	**Decreased release**	**Amperometry** with nomefesine challenge	**None**	**Present**	*Goldberg, 2005*
		DJ-1 KO mouse	**None** at 6-8 week	Single pulse **FCV** +/- Nomefensine	Not investigated	Not investigated	*Sanchez, 2014*
		DJ-1 KO mouse	**None** at 6 months	In vivo **microdialysis** with KCl	**None** by 28 months	**Present**	*Chandran, 2008*
**Risk Factor**	GBA	Wildtype mice with CBE treatment	**Decreased release**	In vivo **microdialysis**	Not investigated	**Present** (data not shown)	*Ginns, 2014*
		iPSC-derived DANs with N370S	**Decreased release**	Evoked with KCl coupled to HPLC-ECD	n/a	n/a	*Woodard, 2014*
